# Apigenin Attenuates the Allergic Reactions by Competitively Binding to ER With Estradiol

**DOI:** 10.3389/fphar.2020.01046

**Published:** 2020-07-16

**Authors:** Lu Yao, Zhuoyan Fan, Shiwen Han, Na Sun, Huilian Che

**Affiliations:** ^1^ Beijing Advanced Innovation Center for Food Nutrition and Human Health, College of Food Science and Nutritional Engineering, China Agricultural University, Beijing, China; ^2^ National Engineering Research Center of Seafood, School of Food Science and Technology, Dalian Polytechnic University, Dalian, China

**Keywords:** apigenin, estrogen receptor, MAPK signaling pathway, Th1/Th2 balance, allergic disease

## Abstract

Apigenin (API) is a natural phytoestrogen with properties including anti-inflammatory and other abilities. This study aims to 1) systematically validate that excessive estrogen exacerbates allergic reactions; 2) explore the anti-allergic effects and mechanisms of API. We conduct a survey of college students, indicating that of the 505 effective results, 70 individuals were self-reported allergic and 74.1% of them were women, which proved the gender difference in allergic reactions. BALB/c mice are grouped into the negative control group (N-Ctrl), the OVA-sensitized group (P-Ctrl), the estrogenized OVA-sensitized group (E2), and three treatment groups administrating different dose of API (E2 + API/L/M/H). *In vivo* data indicated that API treatment significantly inhibited the enhancement of estradiol on clinical symptoms. Moreover, we found that high doses of API inhibited Th2 type humoral response and mast cell degranulation levels *in vivo* and *in vitro*. Additionally, medium, and high doses of API significantly reduced the potentiation of estradiol on ER expression, attenuated the transmission of estrogen/ER signaling, thereby inhibiting the phosphorylation of ERK1/2 and JNK1/2/3 in the MAPK. Besides, we found that API competitively bound to ER with estradiol, and showed a weak selectivity to ERβ. Overall, we identified API can be beneficial in allergic disease.

**Graphical Abstract:**
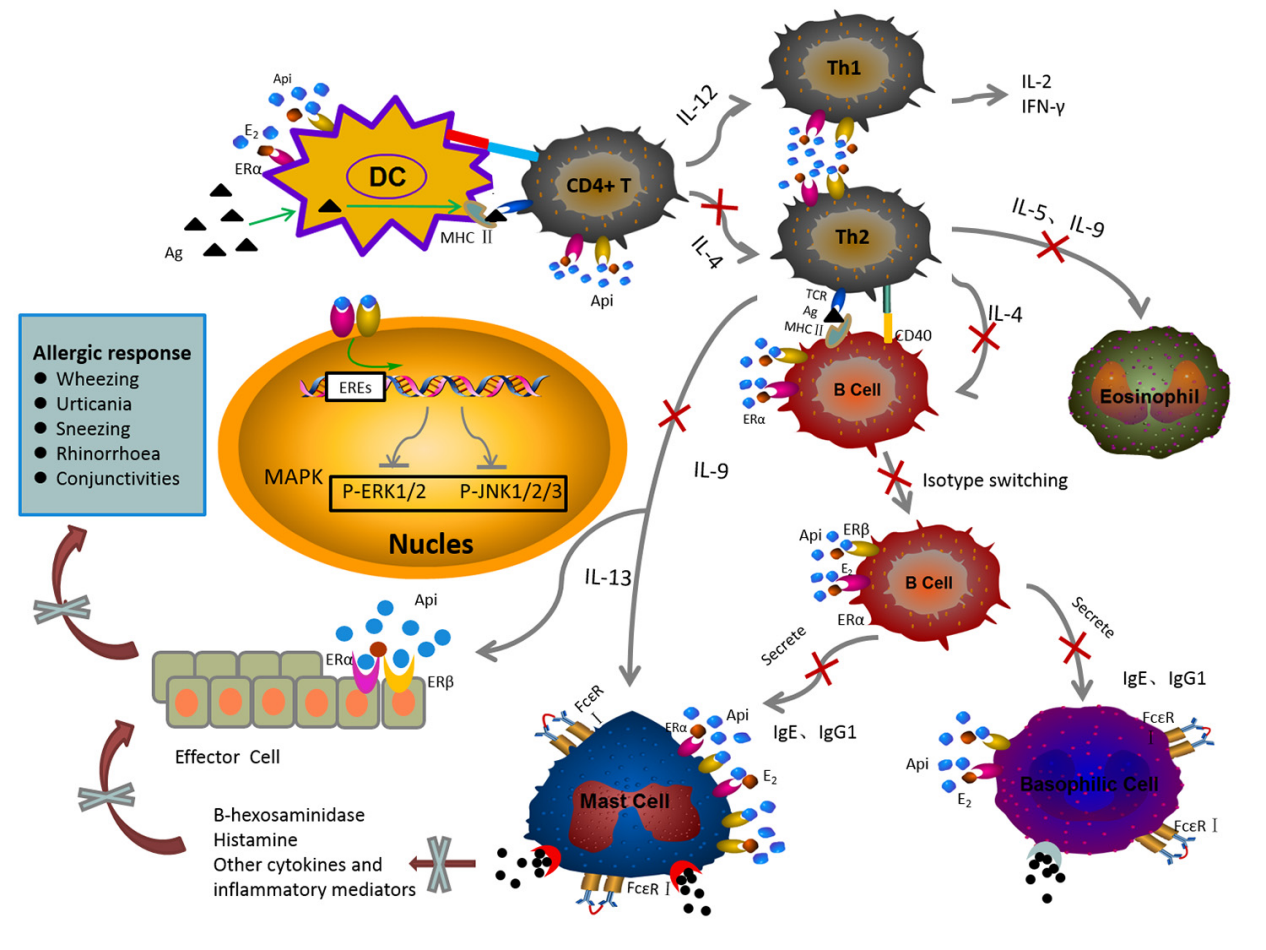
The mechanism of anti-allergic effect of API on the IgE-mediated allergic reaction.

## Introduction

Food allergy refers to a hypersensitivity disorder in which the immune system abnormally reacts to non-infectious environmental substances (Galli et al., 2008), often induces a series of allergic disease complications, such as urticaria, asthma, and diarrhea, which could seriously affect quality of life (Ballegaard et al., 2019). It has been recognized by the World Health Organization (WHO) as a major global food safety issue (Wang et al., 2019) and seriously affected about as many as 10% of children globally (Abrams et al., 2020). Furthermore, its incidence manifests a rising tendency with each passing year (Dupont, 2011). Currently, there are no therapies available to eradicate food allergy completely, although the allergy still occurs frequently (Jiang et al., 2019). In addition, mast cells play important roles in allergy by releasing histamines or inflammatory cytokines after being mediated by IgE (Harvima et al., 2014). The compound which can inhibit mast cell degranulation (Mwakalukwa et al., 2019) or release of inflammatory cytokines is considered to have anti-allergic activity.

In recent years, many data showed that food allergies are observed in higher numbers among women than men (Liew et al., 2009; González-Pérez et al., 2010; Acker et al., 2017; Feuerhake et al., 2018; Wang X-Y et al., 2018), which indicated that women were more susceptible to allergic reactions caused by food than men. Besides, clinical reports have found that the allergic reactions occurring during the menstrual period are cyclical and recurrent (Dorner et al., 2007). Moreover, it has been known for many years that estrogen can affect immune responses (Khan and Ansar Ahmed, 2015). These increasing evidences showed a strongly contribution of sex hormones in the allergic diseases, and it may participate in the susceptibility to allergic reactions.

The estrogen mainly acts as an important immune regulatory factor by binding to ER (Clayton and Wu, 2020). ERs are mainly classified as two classical nuclear estrogen receptors (ERα and ERβ) (Lannigan, 2003; Jordan and Brodie, 2007; Jordan and O’malley, 2007) with a 56% homology between these two isoforms (Powles, 2002). The ERs function as ligand-dependent transcription factors that bind directly to estrogen response element (ERE) in the regulatory region of the estrogen-inducible genes, and then regulate the estrogen responsive gene and playing a series of physiological functions (Lannigan, 2003; Deroo and Korach, 2006; Cunningham and Gilkeson, 2011; Mungenast and Thalhammer, 2014). In addition to the long-term regulation of gene expression, estrogen has also been shown to meditate many rapid biological responses. Researchers have found an estrogen-binding site on the cell membrane (Pietras and Szego, 1975; Pietras and Szego, 1977), and G-protein coupled estrogen receptor (GPER/GPR30) was identified as an estrogen-binding membrane receptor. ERα and ERβ are not only present in the reproductive system but also in the immune cells (Chan et al., 2016). GPER is abundantly expressed in the brain, cardiovascular systems, and the lungs (Feng and Gregor, 1997; Isensee et al., 2009). In addition, previous studies have revealed that the important role of estradiol (E2) and ER signaling on the development and function of B cells, T cells, NK cells, monocytes, and macrophages. And then, these immune cells participate in all stage of the allergic reaction. According to the previous research, administration of E2 could increase the phosphorylated protein expression of ERK through ER signaling. In addition, recent studies suggest that three kinds of MAPK signaling pathways involving extracellular signal-regulated kinases (ERK), c-Jun N-terminal kinases (JNK) and p38 differentially regulate cytokine production (Nakahara et al.). The release of inflammatory cytokines can lead to the breakdown of the Th1/Th2 immune balance, thereby exacerbating the allergic reaction.

Apigenin (4’, 5, 7-trihydroxyflavone), a natural flavonoid, found in a wide variety of dietary plant food such as vegetables and fruits (Rietjens et al., 2017; Madunić et al., 2018). Additionally, the structure of API is similar to E2, which suggesting that it may affect the development of estrogen related disease by altering the level of endogenous estrogen. On the other hand, phytoestrogens compete with endogenous estrogen for binding to ER, which plays an effective role in antagonizing estrogen (Shifren et al., 2010). Previous *in vitro* and *in vivo* studies have found that API has many biological activities while the cytotoxicity is low (Yuan et al.; Zhang et al., 2008). Moreover, many studies have shown that API can regulate the expression of some proteins in the MAPK signaling pathway *in vitro* and *in vivo* (Huang et al.; Lapchak and Boitano, 2014). However, the effect of API on exacerbated allergic reactions due to excessive estrogen levels is unknown. Thus, the aim of the present study was to investigate adverse effects of excessive estrogen on allergic reactions and anti-allergic effect of API from multiple angles *via* ***in vivo*** and ***in vitro*** experiments.

## Materials and Methods

### Reagent

API, E2 were obtained from Sigma-Aldrich (St. Louis, MO, USA). Mouse anti-DNP-IgE monoclonal antibody, DNP-HSA, ovalbumin (OVA), and cholera toxin (CT) were obtained from Sigma-Aldrich. Antibodies for ERα (1:500) and ERβ (1:500) were purchased from Abcam (Burlingame, CA, USA). Mouse IL-4, TNF-α, MCT-1, and histamine ELISA kit were from Dongge Weiye (Beijing, China). Rabbit-derived phosphorylated ERK1/2 monoclonal antibody, rabbit-derived phosphorylated JNK polyclonal antibody, rabbit-derived phosphorylated PLCγ polyclonal antibody was obtained from Lianke Biotechnology (Hangzhou, China). Dulbecco’s modified Eagle medium (DMEM) was purchased from Gibco (Grand Island, NY, USA).

### Mice Models and Treatment

Female BALB/c mice (4 weeks old) weighing 19–22 g in our research were purchased from Vital River Laboratories, Inc. (Beijing, China) and housed in the specific pathogen-free (SPF) animal laboratory of College of Food Science and Nutritional Engineering, China Agricultural University (Beijing, China). Animal rooms were maintained with temperature of 22 ± 1°C, humidity of 55 ± 5%, a 12 h light/dark cycles and air exchanges at 15 times/h. Adaptive feeding for a week, free food and water intake. All animal experiments were performed under the China Agricultural University Animal Experimental Welfare and Ethical Inspection Committee approved protocols and in accordance with ethical standard guidelines of China Agricultural University. All efforts were made to minimize the suffering of experimental animals.

### Estrogenized-Allergic Mice Model

**Figure 1:**
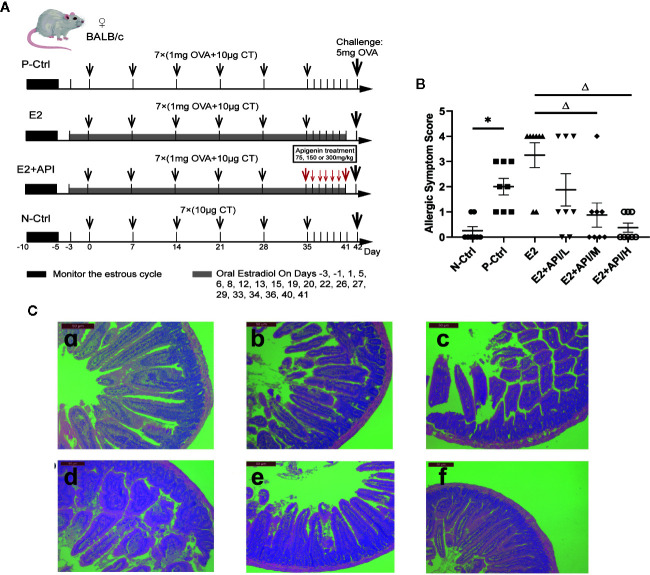
API attenuated clinical allergic symptoms and intestinal injury in BALB/c mice. Histologic analyses were performed on harvested intestinal tissues harvested 1h after challenge on Day 42. **(A)** Schematic drawing representing the BALB/c mice system food anaphylaxis protocols and doses used in this study. **(B)** Allergic symptom score in different group. **(C)** The representative H&E staining picture from jejunum. a: N-Ctrl group; b: P-Ctrl group; c: E2 group (0.15 mg/kg body weight); d–e: E2 + API/L/M/H (75, 150, 300 mg/kg body weight) group. Data are presented as the mean ± SEM from 10 mice. *P <0.05 as compared to the N-Ctrl group, ^△^P <0.05 as compared to the E2 group.

Sixty female BALB/c mice were divided into six group (n = 10) with equal body weight after a week acclimation. The mice in the N-Ctrl group, were administrated orally with 200 µl CT Adjuvant (10 µg in 0.9% NaCl) on days 0, 7, 14, 21, 28, 35 and 42. Mice in the P-Ctrl group, E2 and E2 + API group were sensitized by gavage of 200 µl OVA solution (1mg OVA and 10 µg in 0.9% NaCl) on days 0, 7, 14, 21, 28 and 35. Then, the sensitized mice were challenged intragastrically with a high dose of OVA (5 mg of OVA in 0.9% NaCl) on day 42. Among them, the mice in E2 and E2 + API group were administered orally estradiol solution (0.15 mg/kg E2 in 0.9% NaCl) from 2 days before sensitization to 41 days after the initial sensitization. Before the challenge, API was treated orally at a concentration of 75 mg/kg (low-dose), 150 mg/kg (medium-dose), or 300 mg/kg (high-dose) daily between day 35 and day 41. The specific drug delivery method was shown in **Figure 1A**.

### Clinical Symptom Score

Forty minutes after challenge, mouse systemic allergic symptoms were observed in blinded manner by using a scoring system. The systemic allergic symptoms were measured by clinical allergic symptoms were recorded with reference according to the Li et al. scoring criteria (Li et al., 1999) with scoring specific criteria as followed: 0, no symptoms; 1, nose, lip and eye puffiness; 2, decreased activities; 3, dyspnea, cyanosis, pale around the mouth and tail; 4, convulsions or no response after challenge; 5, death.

### Monitoring Estrus Cycle

All mice were monitored by daily vaginal epithelium cells smear testing during the administration period. The vaginal lavage was fixed with 95% ethanol for 10 min and stained with methylene blue for 10 min (Henry and Witt, 2002). We used the microscopy to observe vaginal epidermal cells. Besides, the keratinized vaginal cells were taken as being indicative of estrus. All estrogenized-allergic mice were checked by daily vaginal epithelium cell smear analysis, in which six consecutive days of constant estrus (**Supplementary Figure 1**).

### Pathological Section and Mast Cell Staining Analysis

After the mice were sacrificed, the small intestine, liver, and kidney were removed and subsequently fixed in phosphate-buffered 10% formalin, and then embedded in paraffin blocks. A section from each paraffin block was stained with hematoxylin and eosin (H&E) to examine the pathologic structures of the tissues. H&E staining of liver and kidney was used to evaluate the safe dose of API (**Supplementary Figure 2**). At the same time, the mast cells of the small intestine of each group were stained with toluidine blue, and the number of mast cells and the degranulation reaction were observed. Images were obtained from fluorescence microscopy.

### RBL-2H3 Cell Culture and Treatment

The rat basophilic leukemia cell line (RBL-2H3) kindly provided from Chinese Academy of Science cell bank was cultured in MEM medium supplemented with 15% fetal bovine serum (FBS) and 1% penicillin/streptomycin/amphotericin B at 37°C in a humidified 5% CO_2_ incubator. Firstly, the cells were cocultured with 1 × 10^−10^ E2 for 4 days in MEM media, and then added to a 6-well culture plate and placed in an incubator for 12 h until cells adhere to the wall. Secondly, cells were preincubated with 1 μg/ml anti-DNP IgE and 1 × 10^−10^ E2 for 2 h. After washing twice with PBS, API at a final concentration of 0, 1 × 10^−9^, 1 × 10^−7^ or 1 × 10^−5^ mol/L and 1 × 10^−8^ mol/L E2 were added to incubate for 45 min. Besides, the cells were preincubated with 10^−7^ mol/L ICI180782 for 1 h at estrogen receptor antagonist group. Finally, the cells were stimulated with 50 μl DNP-HSA (100 ng/ml) for 45 min at 37°C. The specific treatment and groups are listed in the following **Table 1**.

**Table 1:** Treatment groups and doses in RBL-2H3.

Groups	Treatments
**Ctrl**	Firstly, cells were cocultured with 0 mol/L E2 for 4 days in MEM media, and then preincubated with 1 μg/ml anti-DNP IgE for 2 h. After that, 50 μl DNP-HSA (100 ng/ml) were added to stimulated the cells.
**E2**	Firstly, cells were cocultured with 10^−10^ mol/L E2 for 4 days in MEM media, and then preincubated with 1 μg/ml anti-DNP IgE and 10^−10^ mol/L E2 for 2 h. After that, 10^−8^ mol/L E2 were added before stimulating with 50 μl DNP-HSA (100 ng/ml)
**E2 + API/L/M/H**	Firstly, cells were cocultured with 10^−10^ mol/L E2 for 4 days in MEM media, and then preincubated with 1 μg/ml anti-DNP IgE and 10^−10^ mol/L E2 for 2 h. After that, 10^−9^ (L), 10^−7^ (M) or 10^−5^ (H) mol/L API and 10^−8^ mol/L E2 were added before stimulating with 50 μl DNP-HSA (100 ng/ml)
**E2 + API/L/M/H + ICI**	Firstly, cells were cocultured with 10^−10^ mol/L E2 for 4 days in MEM media, and then preincubated with 1 μg/ml anti-DNP IgE and 10^−10^ mol/L E2 for 2 h. After that, the cells were preincubated with 10^−7^ mol/L ICI180782. Lastly, 10^−9^ (L), 10^−7^ (M)or 10^−5^ (H) mol/L API and 10^−8^ mol/L E2 were added before stimulating with 50 μl DNP-HSA (100 ng/ml)

### Cell Viability Assay

Cell viability was measured using Cell Counting Kit-8 (Beyotime Biotechnology, Beijing, China) according to the manufacturer’s instructions. Briefly, 5 × 10^4^ cells were seeded into 96-well plates. After different treatment, 90 μl of medium and 10 μl of CCK-8 solution were added to each well and incubated at 37 °C for 1 h. Absorbance at 450 nm was measured using a Microplate Reader. The results are expressed as a percentage of the control.

### β-Hexosaminidase Release Assay

After stimulation with DNP-HSA, 30 μl of supernatant were transferred to a 96-well plate and incubated with 50 μl of p-Nitrophenyl-N-Acetyl-β-D-Glucosaminide (1.3 mg/ml in 0.1 M citric acid buffer, pH 4.4) for 1 h at 37°C. The reaction was stopped by adding 200 μl stop solution (0.1 M Na_2_CO_3_, pH 9.2). The absorbance of each well was measured at 405 nm using a Thermo Scientific Varioskan Flash microtiter plate reader (Thermo, USA). The total release of β-hexosaminidase was determined in RBL-2H3 cells without E2, API or ICI182780. Besides, the spontaneous release of RBL-2H3 cells was evaluated by adding 50 μl of Tyrode’s buffer instead of DNP-HSA to each well. The release of β-hexosaminidase was calculated as follows (Equation (1)).

(1) $$\begin{array}{c} \beta -hexosaminidase\;release\;(100\%)\\ =\frac{absorbance\;of\;test\;samples\;-absorbance\;of\;Tyrode’s\;solution}{absorbance\;of\;total\;release-absorbance\;of\;Tyrode’s\;solution}\times 100\% \end{array}$$

### OVCAR-3 Cell-Based ER Overexpression Model

The ovarian carcinoma cell line (OVCAR-3) provided from Chinese Academy of Science cell bank was cultured in DMEM medium supplemented with 15% fetal bovine serum (FBS) at 37°C in a humidified 5% CO_2_ incubator. Cells were washed with PBS and then replaced with phenol red-free DMEM (containing 10% CS-FBS) medium to deplete the stored endogenous estrogen 4 days before the treatment. Firstly, we performed plasmid DNA transformation. BL21 competent cells and vector plasmids (pCMV-ERα, pCMV-ERβ plasmid, and pGM-ERE-Luc plasmid) were placed on ice. Then, we combined the competent bacteria with the corresponding plasmid in ice bath for 30 min, heat shock at 42°C for 90 s, and left on ice for 1 min. After that, LB liquid medium were added and shake at 37°C for 1 h, and then let stand for half an hour. Next, we incubated it overnight at 37°C (12–16 h). Secondly, we performed plasmid DNA amplification and plasmid extraction following the instructions of the plasmid extraction kit (Beyotime, Beijing, China). Thirdly, before transfection, OVCAR-3 cells in the logarithmic growth phase were seeded in 12-well plates with no antibiotics, and performed plasmid transfection according to Lipofectamine ™ 3000 Transfection Reagent’s instructions after 24 h. After 24 h of transfection, we extracted the cellular proteins, and detected the expression of two ERs by Western blot.

### Luciferase Reporter Gene Assay

After 24 h of transfection, we removed the culture medium from the 12-well plate, and rinsed the cells twice with PBS. After that, we added a phenol red-free DMEM medium containing 10% CD FBS or a DMEM medium containing 15% FBS and 1 × 10^−10^ mol/L E2 to the 12-well plate. Then, different analytes were separately added and incubated for 48 h. After 48 h, we discard the culture medium. According to the operating instructions of the dual fluorescein detection system kit (Beyotime, Beijing, China), we used a full-wavelength multi-functional microplate reader to measure chemiluminescence.

### ELISA

On the 42th day, mice in each group were bled 45 min after the large stimulation, and centrifuged at 4°C, 5,000 rpm/min to separate the plasma or serum, the stored at −20°C. In addition, the supernatant of different treated cells was transferred to a centrifuge tube, and stored to −20°C. Serum samples which collected from orbital sinus were used to quantified MCT-1, cytokines, estrogen levels, ova-specific IgE, IgG1 and IgG2a using commercial mouse ELISA kit (Abcam, CA, USA). At the meantime, the cell supernatant was used to quantified the levels of histamine, IL-4 and TNF-α using commercial mouse ELISA kit (Abcam, CA, USA).

### Western Blot

Total protein extracts of scraped jejunal mucosa, uterus or cells were harvested by Total Protein Extraction Kit (Hangzhou, China). Equivalent amounts of protein were separated by SDS-PAGE and electroblotted onto PVDF membranes (Beyotime, Beijing, China) followed by blocking with 5% BSA. Then, the membranes were incubated with specific primary antibody at 4°C overnight. Horseradish peroxidase-conjugated secondary antibodies were used to incubated with membranes which were washed with TBST. Protein were visualized by using chemiluminescence reagents and the signal was detected by a gel documentation system (GelDoc-It 310 Imaging System).

### Questionnaire Survey

This is a descriptive cross-section survey based on questionnaire interview. A total of 15 items questionnaire was designed for this survey. The survey questionnaire was divided into two sections. The first section included general characteristics of the total study population such as age, gender, height, and weight. The second section included participant’s knowledge of allergen identification and their own food allergies situation. The survey was conducted in China Agricultural University for students aged 18–22. Additionally, participants had no severe cardiovascular disease, lung disease, kidney disease or metabolic disease. Furthermore, the participants themselves voluntarily participated in this survey. Data were collected in excel sheets. The general characteristics of the study population in food allergy and the type of allergen were respectively shown in **Supplementary Tables 1** and **2**. The allergies of participants and their immediate family members were shown in **Supplementary Table 3**.

### Statistical Analysis

All data were presented as the mean values ± standard error of mean (SEM) from three independent biological replicates. Statistical significance was determined by one-way analysis of variance (ANOVA) using GraphPad Prism 5.01 (GraphPad Software, Inc., USA). A value of P <0.05 was considered to indicate a statistically significant different.

## Results

### API Reversed the Enhancement of E2 on Allergic Response Through the Regulation of T-Helper (Th) Cell Differentiation

According to our previous investigation, among the 505 effective results, 70 individuals were self-reported allergic and 74.1% of them were women, which proved the gender difference in allergic reactions (**Table 2**). In addition, these self-reported allergic people was divided into five categories according to the symptoms, and we found that no matter which type of allergy, there were obvious gender differences, and most of the results showed that women were more susceptible to allergies which provided evidence for this study. To determine whether API inhibited the enhancement of E2 to the allergic response *in vivo*, the estrogenized-allergic model in female BALB/c mice was established. OVA-induced food allergy symptoms were evaluated and scored for allergic symptoms after challenge. Several allergic symptoms of OVA-induced food allergy were observed in the P-Ctrl group, including strongly scratching, decreased activity, dyspnea, cyanosis, pale around the mouth and tail (2 ± 0.327 points) (**Figure 1B**). Furthermore, more severe symptoms including convulsions or no response after challenge were observed in E2 group (3.25 ± 0.491). In contrast, API treatment could suppress the allergic symptoms in a dose-dependent manner (**Figure 1B**). The occurrence of food allergies is closely related to the barrier function of the gastrointestinal tract, so we detected the pathology in small intestine. Compared with the P-Ctrl group, administration of E2 aggregated the intestinal villi loss, mucosal erosion, and a wider distribution of inflammatory cells (**Figures 1C-b, c**). However, different dose of API treatment relieves intestinal lesions to varying degrees (**Figures 1C-d, e, f**).

**Table 2:** Investigation on allergy status of college students.

Gender symptom	Ratio (%)		
	Man	Woman	Total
**Allergic rhinitis**	0.6	1.4	2
**Allergic eczema**	2.6	7.5	10.1
**Allergic gastroenteritis**	0.2	1.2	1.4
**Allergic asthma**	0	0.2	0.2
**Anaphylactic shock**	0.2	0	0.2
**Total**	3.6	10.3	13.9

**Figure 2:**
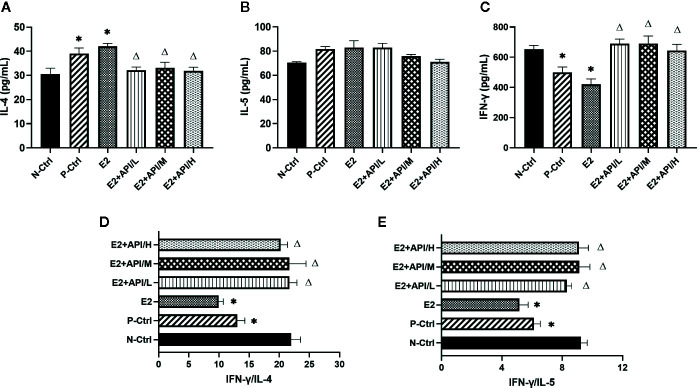
API worked on Th cells to modulate the Th1/Th2 subsets balance. **(A)** Concentration of IL-4, **(B)** IL-5 and **(C)** IFN-γ in serum. **(D)** The ratio of IFN-γ and IL-4, **(E)** IFN-γ and IL-5. Data are presented as the mean ± SEM from 10 mice. *P < 0.05 as compared to the N-Ctrl group, ^△^P < 0.05 as compared to the E2 group.

To further analyze the effects of API and E2 on Th cell differentiation in mice, we also examined the secretion levels of several cytokines. Th2-related cytokine (IL-4/IL-5) was increased by the allergy induction and aggravated by E2 treatment, while administration of API inhibited its level (**Figures 2A, B**). Th1-related cytokine (IFN-γ) decreased in the P-Ctrl group compared to the N-Ctrl group (P <0.05), and this effect was exacerbated by E2 treatment (**Figure 2C**). However, its level significantly increased after API administration (P <0.05, **Figure 2C**). In addition, the results for IFN-γ/IL-4 and IFN-γ/IL-5 was similar to that of IFN-γ (**Figures 2D, E**), and different dose of API ultimately promoted Th1-type immune response. Collectively, our data suggested that API could inhibit Th2-type humoral responses and regulate the Th1/Th2 balance *in vivo*.

### The Level of OVA-Specific Antibodies Produced by B Cell Was Regulated by API

**Figure 3:**
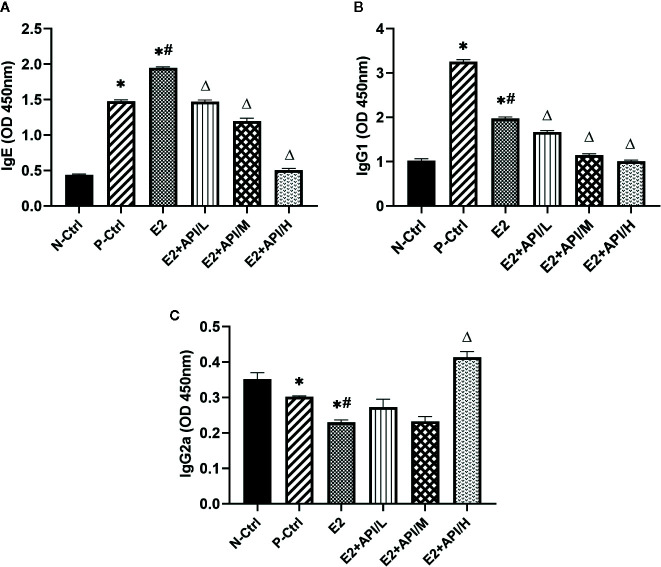
API regulated the production of OVA-specific IgE, IgG1 and IgG2a from B cells. The level of OVA-specific **(A)** IgE, **(B)** IgG1 and **(C)** IgG2a in serum. Data are presented as the mean ± SEM from 10 mice. *P < 0.05 as compared to the N-Ctrl group, ^#^P < 0.05 as compared to the P-Ctrl group, ^△^P < 0.05 as compared to the E2 group.

Next, we examined the levels of serum OVA-specific antibodies by using ELISA assays after different treatments on mouse. The production of IgE and IgG1 was obviously increased in the P-Ctrl group compared to the N-Ctrl group (P <0.05, **Figures 3A, B**). Furthermore, the level of IgE was higher in E2 group, while decreased after API administration (**Figure 3A**). However, we found that the serum IgG1 level was remarkably inhibited after treatment with E2 or API (P <0.05, **Figure 3B**). Interestingly, the significant inhibitory activity of API against the OVA-specific IgE production was similar to its inhibition of the level of IgG1. The above results indicated that administration of API inhibited Th2-type humoral response. On the contrary, the level of IgG2a were found to be decreased in P-Ctrl group, and even lower in E2 group (P <0.05, **Figure 3C**). API treatment had effect on the increase of serum IgG2a level, only high dose administration of API was the most significant (**Figure 3C**). Collectively, these results demonstrated that API influenced OVA-specific antibody-producing B cells and inhibited the Th2-type immune response.

### API Relieved Allergic Symptoms by Suppressing Mast Cell-Mediator Release *In Vivo* and *In Vitro*

**Figure 4:**
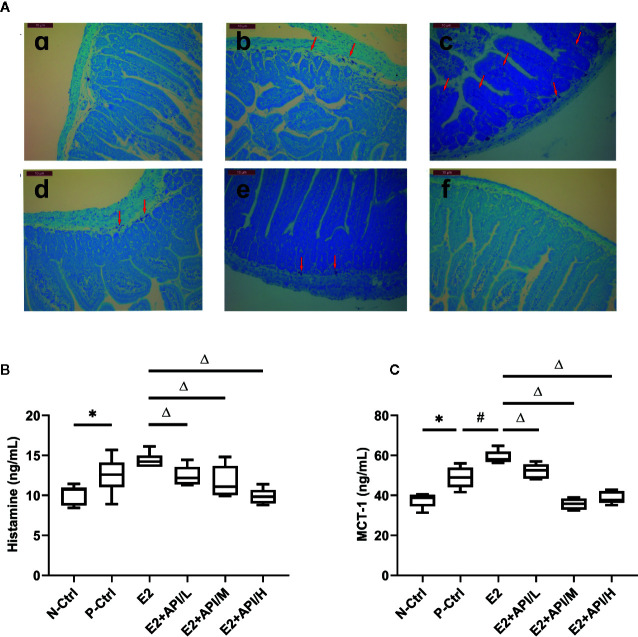
The degranulation of mast cells was attenuated by administration of API *in vivo*. **(A)** Representative Toluidine blue staining results from small intestine tissue of mice, red arrow points to degranulated mast cells. a: N-Ctrl group; b: P-Ctrl group; c: E2 group (0.15 mg/kg body weight); d–e: E2 + API/L/M/H (75, 150, 300 mg/kg body weight) group. **(B)** ELISA analysis results for plasma histamine and serum **(C)** MCT-1. Data are presented as the mean ± SEM from 10 mice. *P < 0.05 as compared to the N-Ctrl group, ^#^P < 0.05 as compared to the P-Ctrl group, ^△^P < 0.05 as compared to the E2 group.

According to previous study, the symptoms of food allergy were caused by the secretion of histamine, MCT-1, β-hexosaminidase and other inflammatory cytokines (Chen et al., 2011). By using estrogenized-allergic animal model and an RBL-2H3 cell-based immunologic assay, we then tested whether E2 can promote mast cell activation and whether API can regulate it. As shown in **Figure 4A**, most of the mast cell aggregation and degranulation occurred in the jejunum tissue of P-Ctrl and E2 group (b, c), and the plasma membrane of the mast cells was blurred. Low-dose API treatment group followed (d). API administration with moderate or high dose significantly inhibited the aggregation and degranulation of the mast cell (e, f). Then, plasma histamine and serum MCT-1 levels were measured using ELISA assays. The levels of histamine and MCT-1 were increased after E2 treatment compared to P-Ctrl group (**Figures 4B, C**). However, administration with API remarkably inhibited its level (P <0.05, **Figures 4B, C**).

**Figure 5:**
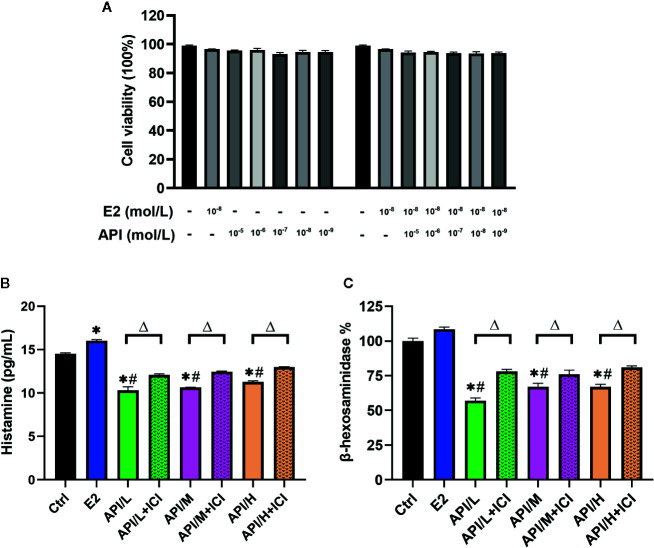
The degranulation of mast cells was attenuated by administration of API *in vitro*. **(A)** CCK-8 cell viability (%) assay for E2 at 1 × 10^−8^ mol/L, API at various concentration (1 × 10^−9^, 1 × 10^−8^, 1 × 10^−7^, 1 × 10^−6^, 1 × 10^−5^ mol/L) or their combination. **(B)** ELISA analysis results for histamine of differently treated cells supernatant. **(C)** β-hexosaminidase release (%) of RBL-2H3 cells after different treatment. Data are presented as the mean ± SEM from 10 mice. *P < 0.05 as compared to the N-Ctrl group, ^#^P < 0.05 as compared to the P-Ctrl group, ^△^P < 0.05 as compared to the E2 group.

Similar results with E2 or API treatment were obtained using the RBL-2H3 cell assay. We first examined the cytotoxic effect of E2 and API on RBL-2H3 cells using the CCK-8 assay and found that they did not affect cell viability (*P >*0.05, **Figure 5A**). Thus, concentrations of 1 × 10^−9^, 1 × 10^−7^ or 1 × 10^−5^ mol/L (API/L/M/H) and 1 × 10^−8^ mol/L (E2) were used for subsequent experiments. To investigate the effect of API on degranulation, we measured the level of histamine and β-hexosaminidase after different treatment. E2 strongly promoted histamine (P <0.05) and β-hexosaminidase release (**Figures 5B, C**). On the contrary, data demonstrated that API significantly suppressed the level of histamine and β-hexosaminidase (**Figures 5B, C**). Furthermore, the β-hexosaminidase release was reduced by up to 51.50% ± 1.443% after administration of 1 × 10^−5^ mol/L API (**Figure 5C**). This effect of API was eliminated after ICI 182780 treatment which indicating that the API may work through ERα or ERβ. Taken together, these results demonstrated that API inhibited the promotion of E2 on mast cell degranulation through ERα or ERβ.

### The Effect of E2 on ERα and ERβ Was Attenuated by API *In Vivo* and *In Vitro*

**Figure 6:**
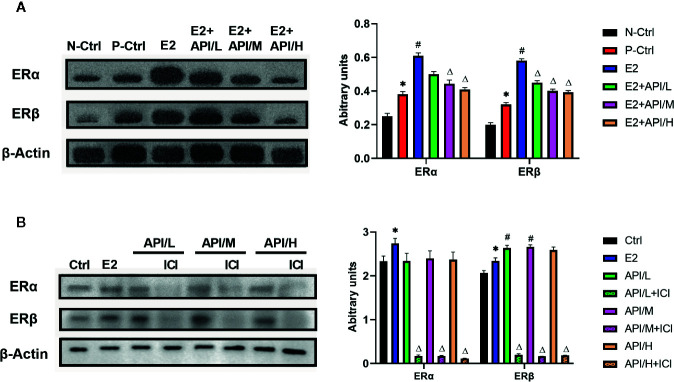
API treatment reduced the enhancement of E2 on ER expression *in vivo* and *in vitro*. Collecting cells after degranulation and extracting the total protein. **(A)** Western blot analysis on the expression level of ERα and ERβ from respective uterus tissues and **(B)** the protein of cells. The panel shows the relative levels quantified by densitometry and normalized to β-actin, and data are presented as the mean ± SEM from 10 mice. *P < 0.05 as compared to the N-Ctrl group, ^#^P < 0.05 as compared to the P-Ctrl group, ^△^P < 0.05 as compared to the E2 group.

To explore the effects of API and E2 on the expression of ER in target tissue, we established western blot analysis to investigate the expression level of ERα and ERβ in uterus tissue. Results showed that ERα and ERβ expression were significantly increased after the treatment of E2, and API administration attenuated this effect in a dose-dependent manner (**Figure 6A**). Then, in order to investigate this effect, the estrogenized mast cell model was established in RBL/2H3 cell. As shown in **Figure 6B**, E2 significantly up-regulated the expression level of ERα, which increased the expression of ERβ. After treatment with API, the expression level of ERβ in RBL-2H3 cells was significantly increased, but there was no significant effect on the expression level of ERα. All in all, these results suggested that API decreased the enhancement effect of E2 on estrogen receptor expression, further indicating estrogen receptor was a target of API.

### API Inhibited MAPKs Signaling Pathway Through ER, Thereby Reducing the Secretion of Allergic Inflammatory Cytokine

**Figure 7:**
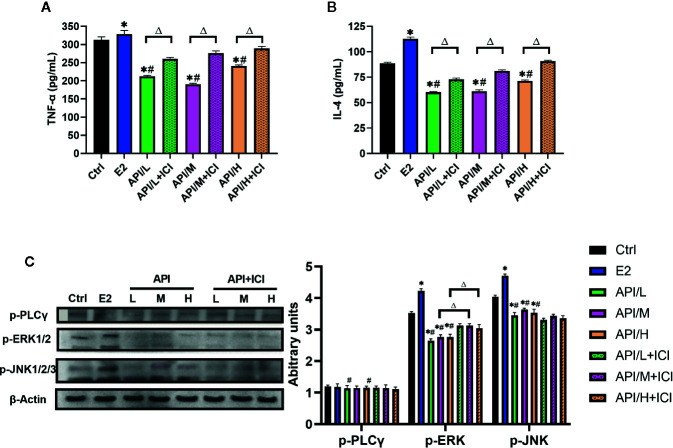
API reduced the secretion of allergic inflammatory cytokine through MAPKs signaling pathway. **(A)** Concentration of TNF-α, **(B)** IL-4 in the cell supernatant after different treatment. **(C)** Western blot analysis on the expression level of p-ERK1/2, p-JNK1/2/3 and p-PLC γ. The panel shows the relative levels quantified by densitometry and normalized to β-actin, and data are presented as the mean ± SEM from 10 mice. *P < 0.05 as compared to the N-Ctrl group, ^#^P < 0.05 as compared to the P-Ctrl group, ^△^P <0 .05 as compared to the E2 group.

In order to explore the mechanism of API inhibiting mast cell degranulation and inflammatory cytokine secretion, we established ELISA assays to measure the level of histamine (**Figure 5B**), β-hexosaminidase (**Figure 5C**), IL-4 and TNF-α. Our results showed that these cytokines were significantly increased after the treatment of E2, and API administration attenuated this effect (**Figures 7A, B**). Besides, ICI 182780 administration could reverse the effect of API in a way. Next, the expression level of PLC γ, ERK1/2 and JNK1/2/3 were investigated to determine whether the expression change of ER caused by API could inhibit MAPK signaling pathway or PLC-γ phosphorylation. After treated with API, phosphorylated protein expression levels of ERK1/2 and JNK1/2/3 were significantly decreased compared with E2 treatment group, while expression of p-PLC γ was decreased in individual groups (**Figure 7C**). In addition, we also observed that treatment of RBL-2H3 cells with ICI 182780 increased the phosphorylation levels of ERK1/2 (**Figure 7C**). These finding suggested that API inhibited the activation of MAPKs signaling pathway through ER and inhibits inflammatory cytokine release.

### API Regulated ERE Luciferase Activity Bidirectionally and Competed With E2 for Binding to ER, Showing Weak Selectivity for ERβ

**Figure 8:**
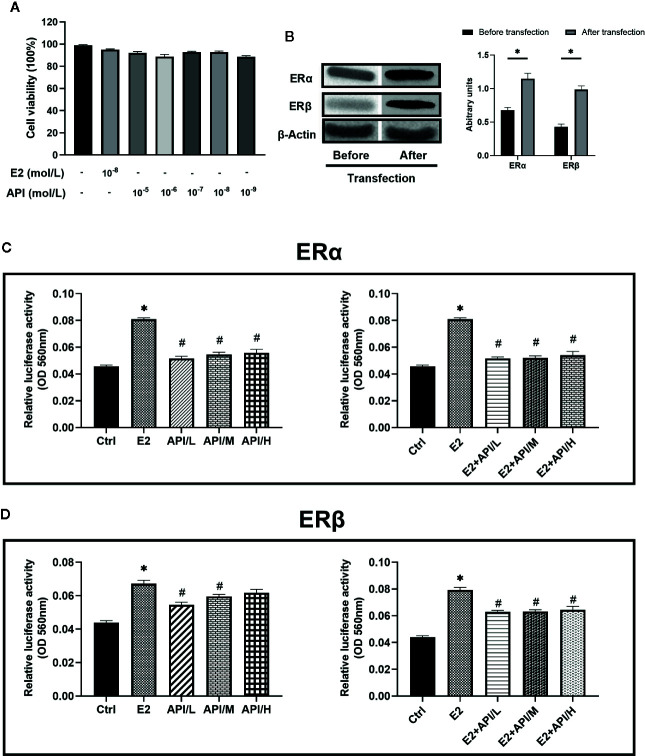
API regulated ERE luciferase activity bidirectionally and weakly competed with E2 for binding to ERβ. **(A)** CCK-8 cell viability (%) assay for E2 at 1 × 10^−8^ mol/L and API at various concentration (1 × 10^−9^, 1 × 10^−8^, 1 × 10^−7^, 1 × 10^−6^, 1 × 10^−5^ mol/L). **(B)** Western blot analysis on the expression level of ERα and ERβ. **(C, D)** OVCAR-3 intracellular ERE luciferase activity showed the ability of API to bind to estrogen receptor and its estrogenic antagonist activity. The panel shows the relative levels quantified by densitometry and normalized to β-actin, and data are presented as the mean ± SEM from 10 mice. *P < 0.05 as compared to the Ctrl group, ^#^P < 0.05 as compared to the E2 group.

To assess the binding activity of API or E2 to ER (ERα and ERβ), we conducted luciferase reporter gene experiments based on OVCAR-3 cells. In order to investigate the effect of API and E2 on the cell viability of OVCAR-3 cells, CCK-8 assay was established. OVCAR-3 cells were first treated with different dose of API or E2, and we found that they did not affect the cell viability (P >0.05, **Figure 8A**). Therefore, concentrations of 1 × 10^−9^, 1 × 10^−7^ or 1 × 10^−5^ mol/L (API/L/M/H) and 1 × 10^−8^ mol/L (E2) were used for subsequent experiments. Then, we transfected two ER plasmids into OVCAR-3 ovarian cancer cells, and used Western Blot analysis to monitor the expression of ERα and ERβ. After transfection, the level of ERα and ERβ was remarkably increased in OVCAR-3 cells (**Figure 8B**). As shown in **Figures 8C, D**, administration of E2 significantly upregulated the activity of ERE luciferase in OVCAR-3 cells (P <0.05). Moreover, E2 was more selective for ERα than ERβ. Meanwhile, different doses of API also increased the ERE luciferase activity (P <0.05). Consistent with previous research, some phytoestrogen compounds have lower affinity for ERs compared to E2 (Collins-Burow et al., 2000), the same to API (**Figures 8C, D**). To detect estrogen antagonistic activity of API, cells were co-treated with E2 and API at the same time. To our surprise, we found that API treatment could significantly down-regulate the effect of E2 on ERE luciferase activity. Besides, administration of API significantly reduced the binding activity of E2 to ERβ (**Figure 8D**) compared to ERα (**Figure 8C**), which indicating that API had a weak selectivity for ERβ. This ability might be one of the reasons for the different effect between API and endogenous estradiol. In conclusion, these results proved that API competitively bound to ERα and ERβ with E2 and show a selectivity for ERβ, then exerted the corresponding effects.

## Discussion

API has been shown to have diverse biological activities, such as anti-oxidation, anti-inflammatory, and anti-angiogenesis activities (Melstrom et al., 2011; Patil et al., 2015; Cao et al., 2016; Patil et al., 2016; Xu et al., 2016). In this study, the anti-allergic activity of API was confirmed. Our study demonstrated that excess E2 induced the enhancement of allergic reactions *in vivo* and *in vitro*, while API showed the opposite effects. As shown in GRAPHICAL ABSTRACT, the main mechanisms of anti-allergic effect of API are summarized as followed: API (1) regulates the Th cell differentiation, which decreased the elevated level of Th2-related cytokine to restore Th1/Th2 immune balance; (2) affects the OVA-specific antibody producing by B cells; (3) inhibits the degranulation of mast cells and the release of inflammatory cytokines through inhibiting MAPK signaling pathway by competitively binding ER with E2. This research provided a scientific basis for meeting the urgent need to find new drug sources and effectively way to prevent food allergic diseases.

Activation and differentiation of T cells exerts a critical role in the allergic disease, and CD4^+^T cells differentiate into Th1 cells and Th2 cells. Th2 cells are the major Th cell subset involved in the hypersensitivity sensitization phase (Eigenmann, 2009; Shin et al., 2015; Voskamp, 2014), and they are essential to the secretion of Th2 cytokines. In an allergic reaction, Th2 cells secrete a large number of inflammatory cytokines, thereby recruiting effector cells such as mast cells, leading to an imbalance of Th1/Th2. These pro-inflammatory cytokines are important mediators of inflammation (Wang et al., 2017). Previous studies have shown that estrogen can change the phenotype and function of lymphocyte and change the body’s immune response *via* affecting the synthesis and secretion of cytokines (Hasegawa et al., 2006; Grimaldi et al., 2002). When estrogen acts on the immune system, it may affect the body’s immune function by regulating the dynamic balance between Th1/Th2 (Hong et al., 2014; Ngo et al.). Actually, the role of estrogen in immunity has been extensively reviewed (Gubbels Bupp et al., 2018). In addition, many reports have identified that API can affect the secretion of cytokines by directly affecting Th cell function. In an mice model of experimental autoimmune myocarditis (EAM), researcher found that the generation of IFN-γ was significantly increased, while the generation of IL-4 was markedly declined compared with the control group (Zhang et al., 2016). (4) The result is like the previous findings (Patil et al., 2016; Wang F et al., 2018), which points to many biological roles of API including suppressing the stimulation of T-cell proliferation and the differentiation of Th2 cells. The results of above researches are consistent with the present study, which identified the modulatory effect of API on Th cells. Moreover, our data indicated that API could regulate the balance of Th1/Th2.

In addition to regulated the Th1/Th2 immune balance, administration of API can significantly suppress the IgE levels in mice (Yano et al., 2006). In an allergic asthma mouse model, treatment with API reduced total serum IgE levels to near baseline compared with the OVA group (Li et al., 2010). In another study, dietary API significantly alleviated the development of skin lesions, accompanied by lower serum IgG1 and IgE levels in NC/Nga mice (Yano et al., 2009). The Specific IgG antibodies also play a crucial role in the pathogenesis of allergic diseases. Our research found that treatment of E2 aggravated the allergic reaction, which was manifested by the increase of Th2 antibody (IgE and IgG1) and the decrease of Th1 antibody (IgG2a). Similar to previous studies, we found that API treatment reduced the level of Th2 antibody and increased the level of Th1 antibody.

Previous studies have found that higher numbers of mast cells have been observed in the mammary glands of female rats in association with higher histamine levels during the estrous than in proestrus or diestrus stage (Jing et al., 2012). It could be seen that changes in estrogen levels are related to mast cells releasing inflammatory substances. In addition, early researches have indicated that API can inhibit the release of histamine (Ogasawara et al., 1986). In our present study through the mast cell toluidine blue staining, exogenous administration of E2 will promote mast cell degranulation, while treated with different concentrations of API could attenuate the degranulation of mast cells. Similarly, API can suppress the release of histamine and β-hexosaminidase, the biomarker of degranulation in RBL-2H3 cells. When using classic estrogen receptor inhibitors (ICI182780), the effects of API were eliminated to some extent. Our data indicated that excessive E2 stimulated mast cell activation and degranulation, while API treatment inhibited this effect through ER signaling.

Estrogen plays its role mainly by binding with ERs (ERα and ERβ). ERs are widely distributed in immune cells (Straub, 2007; Cunningham and Gilkeson, 2011), so estrogen modulates the function of immune cells involved in allergic reactions, thus playing an important role in every phase of allergic reactions. In recent years, several studies have revealed the potential relation between API and ERs (ERα and ERβ) (Mak et al., 2006; Meng et al., 2017). In a cell assay, data showed that API increased estrogen-regulated gene transcription in Hela cells transiently transfected with ERα (Miksicek, 1994). The above results provided evidence for API to regulate estrogen/ER signaling. Our research indicated that administration of API reduced serum E2 levels in a dose-dependent manner (**Supplementary Figure 3**). In this study, we found that API could inhibit ER expression *in vivo*, but *in vitro* experiments did not show this result. ER was found in many organs throughout the body, and the decrease expression of ER in the uterus did not fully explain that API would reduce the expression of all ER in the body. *In vitro* data of the estrogenized mast cell model, our results found that ERβ expression was elevated. These finding hinted that ERβ may play a good role in allergic reactions. Besides, GPER, which differs in signaling mechanisms, expression, binding affinity to estrogen and biological functions from the classical nuclear ERs (ERα and ERβ), should also be considered. Recent studies established the important roles of GPER as a novel membrane receptor for estrogen (Tamaki et al., 2014). In addition to the effect of GPER in diverse pathological and physiological events including cancer and the reproductive, nervous, and cardiovascular systems, its function in the immune system has gradually emerged (Prossnitz and Barton, 2011). However, the role of GPER in allergic inflammation is poorly understood. We also don’t know whether API affect the process of food allergy by regulating GPER expression. This will be one of the research directions in future.

For the balance mechanism of API to regulate Th1/Th2 immune response, we conducted in-depth discussion. The most direct manifestation of allergic reactions is degranulation, release, and synthesis of bioactive mediators of mast cells. Then, there are two main ways to induce degranulation of mast cells. One is γ-chain isomerism phosphatidylinositol-specific phospholipase C (PLC γ) activation. PLC γ-dependent increase in cytosolic free calcium and PKC activation are necessary signals for degranulation (Kassessinoff et al., 1993). Another pathway is activation of mitogen-activated protein (MAP) kinase. MAPK extracellular signal-regulated kinase 1 (ERK1), ERK2, p38 and JNK activate transcription factors activate T cell nuclear factor (NFAT) and nuclear factor kappa B, leading to cytokine production (Kim et al.). Therefore, MAPK may be an effective target for the treatment of allergic inflammation. On the other hand, MAPK signaling pathways also play an essential role in the regulation of the release of cytokines (Che et al., 2019). Activation of MAPK signaling pathway protein leads to increased release of inflammatory cytokines such as IL-1β, IL-6, and TNFα (Zarubin and Jiahuai, 2005; Che et al., 2019). Therefore, MAPK become a major target for the treatment of inflammatory and allergic diseases. In the present study, we found that E2 significantly enhanced phosphorylation of ERK1/2 and JNK1/2/3 after FcϵRI cross-linking, whereas API treatment significantly reduced their phosphorylation levels, suggesting that API attenuated the secretion of cytokines by inhibiting MAPKs signaling pathway. In contrast, E2 and API have a reduced effect on PLC-γ phosphorylation in RBL-2H3 cells. We speculated that API may act primarily on downstream signals of p-PLCγ, thereby inhibiting mast cell degranulation and the release of inflammatory cytokines. In summary, as confirmed by estrogenized allergic mice model and RBL-2H3 cell-based immunology assay, API could effectively attenuate the enhancement of the severity of food allergy induced by excess E2, and the underlying mechanism might be associated with the modulation of ER/MAPK signaling.

## Data Availability Statement

The raw data supporting the conclusions of this article will be made available by the authors, without undue reservation, to any qualified researcher.

## Ethics Statement

The studies involving human participants were reviewed and approved by: The ethical standard guidelines of China Agricultural University. The patients/participants provided their written informed consent to participate in this study. The animal study was reviewed and approved by China Agricultural University Animal Experimental Welfare and Ethical Inspection Committee.

## Author Contributions

Conceptualization: LY, HC. Methodology: LY, ZF, HC. Investigation: SH, NS. Data curation: LY, ZF. Writing—original draft: LY, HC. Writing—review and editing: LY, ZF, and HC. Funding acquisition: HC. Resources: HC.

## Funding

This research was supported by National Natural Science Foundation of China under grant No. 81773435.

## Conflict of Interest****

The authors declare that the research was conducted in the absence of any commercial or financial relationships that could be construed as a potential conflict of interest.
