# Supplementary material for: Apigenin Attenuates the Allergic Reactions by Competitively Binding to ER With Estradiol
Source: Front Pharmacol. 2020 Jul 16;11:1046. doi: 10.3389/fphar.2020.01046 (PMC7378534; doi:10.3389/fphar.2020.01046)
Supplement: Supplementary file 1 [file DataSheet_1.docx]

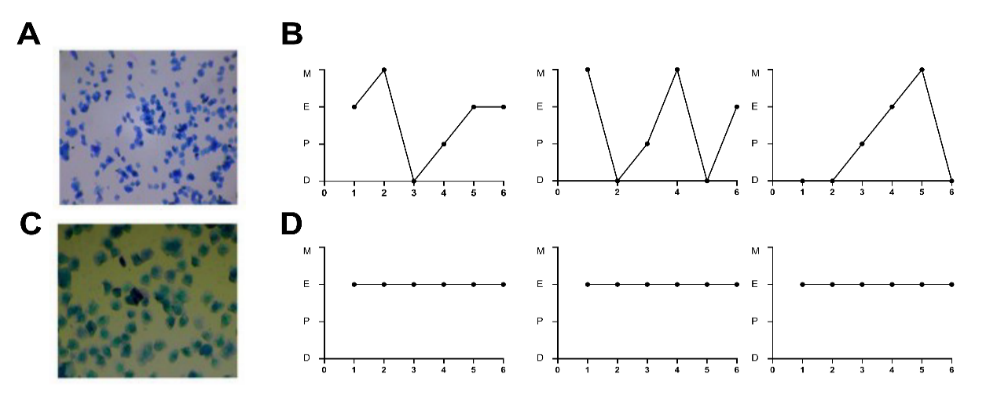


**Supplementary Figure 1** Effects of apigenin (API) on estrous cycle patterns of mice. Vaginal epithelium cell smears were taken at the seventh day from different group. (A) The picture of vaginal epithelium cell smears in control mice. (B) The estrous cycle of three representative mice in control mice. (C) The picture of vaginal epithelium cell smears in estrogenized mice. (D) The estrous cycle of three representative mice in estrogenized mice.


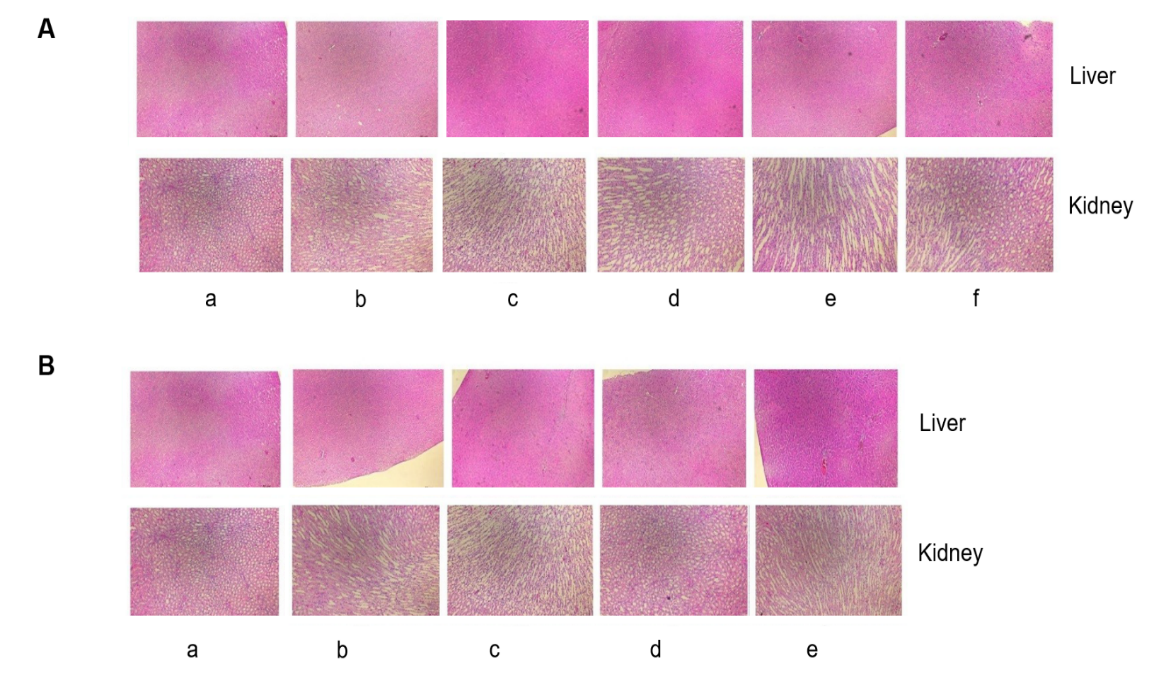


**Supplementary Figure 2** Representative H&E staining pictures of liver and kidney. Representative photomicrographs taken at 50× magnification of liver and kidney sections from each treatment group. The H&E staining picture of liver and kidney in estrogenized mice. a: N-Ctrl group; b: P-Ctrl group; c: E2 group (0.15mg/kg body weight); d-e: E2+API/L/M/H (75mg/kg, 150mg/kg, 300mg/kg body weight) group.


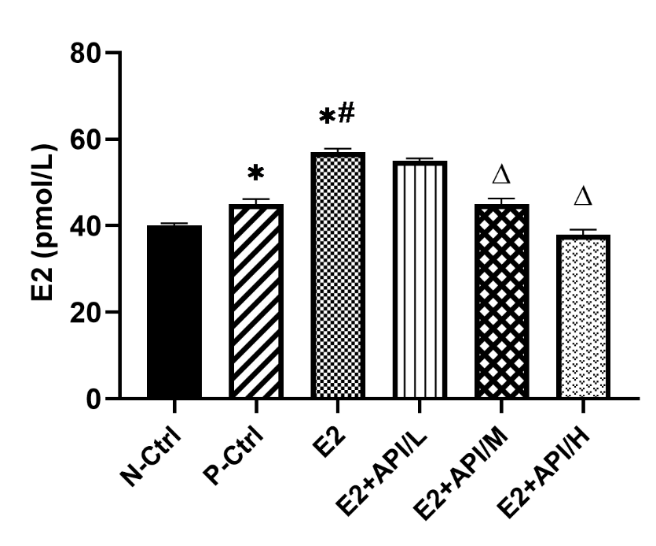


**Supplementary Figure 3** Concentration of E2 in serum. Data are presented as the mean ± SEM from 10 mice. *P <0.05 as compared to the N-Ctrl group, #P <0.05 as compared to the P-Ctrl group, △P <0.05 as compared to the E2 group.

**Supplementary Table 1** General characteristics of the study population in food allergy

| **Characteristic** | **Number of people (n=70)** | |
| --- | --- | --- |
|  | **No** | **Yes** |
| Sex (male) | 52 | 18 |
| BMI (kg/m^2^, 18.5-25) | 22 (female: 15) | 48 |
| Nation (Han) | 2 | 68 |
| Eight categories of allergens | 43 | 49 |
| Diagnosis | 25 | 45 |
| Immediate family | 10 | 60 |
| First food allergy (13-18 years old) | 10 | 60 |
| Antibiotic frequency (<5 times) | 2 | 68 |

**Supplementary Table 2** The types of allergen

| **Allergen** | **Numbers (n=70)** | **Frequency (%)** |
| --- | --- | --- |
| Crustaceans | 29 | 39.7% |
| Mango | 18 | 24.7% |
| Peach | 6 | 8.2% |
| Jujube | 2 | 2.7% |
| Other fruits | 8 | 11% |
| Milk | 6 | 8.2% |
| Fish | 8 | 11% |
| Eggs | 4 | 5.5% |
| Nuts | 2 | 2.7% |
| Beef or mutton | 2 | 2.7% |
| Chives | 2 | 2.7% |
| Alcohol | 2 | 2.7% |
| Unknown food | 3 | 4.1% |

**Supplementary Table 3** Allergies of participants and their immediate family members

| **Viability** | **Frequency (%)** | |
| --- | --- | --- |
|  | **Yes** | **No** |
| Did your immediate family members (parents or siblings) cause allergies after contact or inhalation of a substance? | 20.4 | 79.6 |
| Did your immediate family member (parent or sibling) cause an allergy after eating a certain substance? | 11.9 | 88.1 |
| Do you cause food allergies after contact or inhalation of a substance? | 21.8 | 78.2 |
